# The influence of kinematic viscosity of oils on the energy consumption of a gear pump used for pumping oil in machines and vehicles

**DOI:** 10.1371/journal.pone.0331371

**Published:** 2025-09-02

**Authors:** Łukasz Warguła, Łukasz Gierz, Olga Zharkevich, Bartosz Wieczorek, Łukasz Wojciechowski, Karolina Perz, Alexandra Berg, Andrey Berg, Darkhan Zhunuspekov, Asset Altynbaev, Piotr Kaczmarzyk, Anna Dziechciarz

**Affiliations:** 1 Institute of Machine Design, Faculty of Mechanical Engineering, Poznan University of Technology, Poznań, Poland; 2 Department of Technological Equipment, Mechanical Engineering and Standardization, Abylkas Saginov Karaganda Technical University, Karaganda, Kazakhstan; 3 Institute of Machines and Motor Vehicles, Faculty of Civil Engineering and Transport, Poznan University of Technology, Poznań, Poland; 4 Scientific and Research Centre for Fire Protection, National Research Institute, Józefów, Poland; CINVESTAV IPN: Centro de Investigacion y de Estudios Avanzados del Instituto Politecnico Nacional, MEXICO

## Abstract

The general theory of oil pumping using gear pumps shows that as kinematic viscosity increases, so does the energy requirement to drive the pump shaft. However, modern oils used in machines and vehicles are characterized by a wide range of modifiers that alter their physical and chemical properties. This article presents a study on the energy demand for driving a gear pump while pumping commercial oils used in machines and vehicles (16 types), such as those for combustion engines in single-drive and hybrid vehicles, gearboxes, hydraulic systems, shock absorbers, chainsaw lubrication, and two-stroke engine fuel mixtures. For the tested oils, kinematic viscosity was determined at 25°C and 50°C, and the mechanical power required to pump them at an ambient temperature of approximately 25°C was measured. Based on the measured power and rotational speed, the energy demand for driving the gear pump was calculated. The main analysis was conducted under recommended operating conditions, where the pump operates most efficiently—namely, at a rotational speed of 2000 rpm and a pressure of 20 MPa. It was shown that within the tested group, kinematic viscosity is not the primary factor determining the energy intensity of the oil pumping process. However, this relationship becomes more evident when oils are grouped by application. The energy consumption during pumping of oils with kinematic viscosity at 25°C ranging from 21 to 784.5 mm^2^/s varies from 606 to 734 Wh. The difference due to the type of oil is approximately 21%. The lowest energy consumption was observed for the HL 46 hydraulic oil, which, although not having the lowest kinematic viscosity, was specifically designed by the manufacturer for use with gear pumps—indicating that pump designs are tailored to the specific type of oil being pumped.

## 1. Introduction

The transport of liquids using gear pumps refers to the process of moving liquids through specialized devices known as gear pumps [1]. These pumps utilize rotating gears to transfer fluid within the system. Gear pumps consist of two intermeshing gears. As the gears rotate, liquid is drawn into the pump on the inlet (suction) side due to the vacuum created in the spaces between the teeth. The liquid is then moved around the perimeter of the gears, and the teeth carry it toward the outlet. At the outlet, the gears interlock, forcing the liquid out under pressure [2,3].

Gear pumps are widely used in the transport of liquids, particularly those with high viscosity, such as oils [4], fuels [5], crude oil [6], chemicals [7], and lubricants [8]. The main characteristics of this process include precision and flow control, high pressure resistance, and adaptability to various liquid viscosities. Gear pumps offer a consistent and controlled flow, which is crucial in industrial processes. They are capable of operating under high pressure, enabling the pumping of liquids over long distances or through systems with high flow resistance. Gear pumps are well-suited for handling liquids with varying viscosities, making them a versatile solution in many applications. Thus, gear pumps are a critical component in fluid transport systems across various industries, offering reliability, durability, and energy efficiency [9].

Oils during transportation (pumping) differ in their flow resistance in pumps [10]. The classification of oils based on pumping resistance is primarily related to their viscosity and rheological properties, which affect the performance of hydraulic systems, lubrication, and fuel pumps. In this context, oils can be divided into several basic categories: low-viscosity oils [11], medium-viscosity oils [12], and high-viscosity oils [13]. Additionally, multi-viscosity oils [14], synthetic oils [15], and specialized oils, such as biodegradable oils [16,17] or high-pressure resistant oils [18], can also be distinguished.

Low-viscosity oils have a low viscosity, meaning they flow easily through the system but provide weaker lubrication under higher loads and temperatures [19]. They are used in systems requiring rapid fluid flow and minimal resistance, such as in certain hydraulic systems and the lubrication of precision mechanisms.

Medium-viscosity oils offer a balance between ease of flow and the ability to form a lubricating layer. They are most commonly used in pumps and hydraulic systems where moderate pumping resistance and good protection of mechanisms are required [20]. Typical examples include hydraulic oils with ISO VG 32, 46, or 68 viscosity grades.

High-viscosity oils are characterized by high viscosity, resulting in greater pumping resistance, but they provide effective lubrication and protection at high temperatures and under heavy loads [21]. These oils are used in systems requiring significant pumping forces and mechanical loads, such as industrial pumps, gear systems, and heavy equipment lubrication. Examples include gear oils, high-viscosity lubricants, and hydraulic oils with ISO VG 100 or higher.

Multi-viscosity oils are special oils whose viscosity changes depending on the temperature, enabling them to provide lower resistance at low temperatures and good protection at high temperatures. These oils are used in systems that operate across a wide range of temperatures, such as hydraulic and engine systems in vehicles. Examples include multi-season engine oils (e.g., 5W30, 10W40) and high viscosity index (HV) hydraulic oils.

Synthetic oils, due to their chemical structure, exhibit better stability at high temperatures, resistance to aging, and improved fluidity at low temperatures, which can reduce pumping resistance. They are used in systems that require high resistance to temperature fluctuations and long-term operation, such as in aviation, automotive applications, and modern hydraulic systems. Examples include PAO (polyalphaolefin) synthetic oils and ester-based oils.

Specialized oils, such as biodegradable oils and high-pressure resistant oils, can be developed to reduce resistance under specific working conditions, such as high pressures, extreme temperatures, or environments requiring biodegradable substances [22]. They are used in systems with specific environmental requirements or extreme working conditions. Examples include biodegradable oils and HFC (high-pressure-resistant synthetic) oils.

Each type of oil is selected based on the specific needs of a hydraulic or lubrication system, considering factors such as viscosity, operating temperature [23,24], load, and specific operational requirements. In industrial and commercial processes, oils often require transportation, for instance, for distribution into tankers or smaller containers. However, due to the physical properties of oil, this process can influence its energy consumption. One of the fundamental physical parameters of oil is its kinematic viscosity, which has a significant impact on the flow resistance in a gear pump, as viscosity determines how easily the liquid can flow through the system. In the context of gear pumps, higher kinematic viscosity can increase flow resistance, thereby affecting the pump’s efficiency.

The aim of this paper is to determine the impact of the type of oil used in vehicles and machinery, with varying kinematic viscosities, on the energy demand of a gear pump. The study examines the drive power on the pump’s input shaft during the pumping of combustion engine oils [25], hydraulic oils [26], gear oils [27,28], as well as oils used in shock absorbers [29], air conditioning systems, fuel chain lubrication in internal combustion engines [30], and in two-stroke engines with a mixture-lubricated ignition system [31]. Estimating energy consumption based on power measurements involves calculating the amount of energy used by a device or system based on its power output and operating time [22].

## 2. Materials and methods

The energy consumption during the pumping of oils was determined based on the measurement of mechanical power *P* on the input shaft of the gear pump. The installation of a torque meter (Roman Pomianowski Electronics Studio, Poznań, Poland) for measuring torque *M*, equipped with a function for measuring rotational speed *n* between the drive unit and the gear pump, enabled the calculation of mechanical power *P* using the following formula (this measurement method was also employed in the study of other machines [32–34]):


P=M·ω

where:

*ω* is the angular velocity in radians per second (rad/s), which is calculated from the rotational speed *n* using the formula:


$$\omega =\frac{2\cdot \pi \cdot n}{60}$$

Hence the final formula for mechanical power is:


$$P=M\cdot \frac{2\cdot \pi \cdot n}{60}$$

To determine energy consumption based on mechanical power measurement, the following relationship was used: mechanical energy *E* is the product of mechanical power *P* and working time *t*:


E=P·t

The applied hydraulic gear pump symbol 1.40.09.00.105 manufactured by Agricola Hydraulika Siłowa Sp. z o. o. Sp. k. from (Lubień Kujawski, Poland) is used universally, in small and medium-sized mobile systems, power packs, power supplies, aggregates, car lifts, in hydraulic systems according to proprietary designs and others, the pump characteristics are presented in Table 1.

**Table 1:** Characteristics of the pump.

Parameter	Value	Unit
Pump type	Gear pump	–
Maximum flow rate	1.65 (at 1500 rpm)	L/min
Geometric displacement	1.10	cm³/rev
Rotational speed range	600 - 6000	rpm
Connections	Internal threads 3/8“	–
Recommended maximum continuous operating pressure	20	MPa
Maximum pressure	25	MPa
Recommended rotational speed for continuous operation	2000	rpm

**Fig 1:**
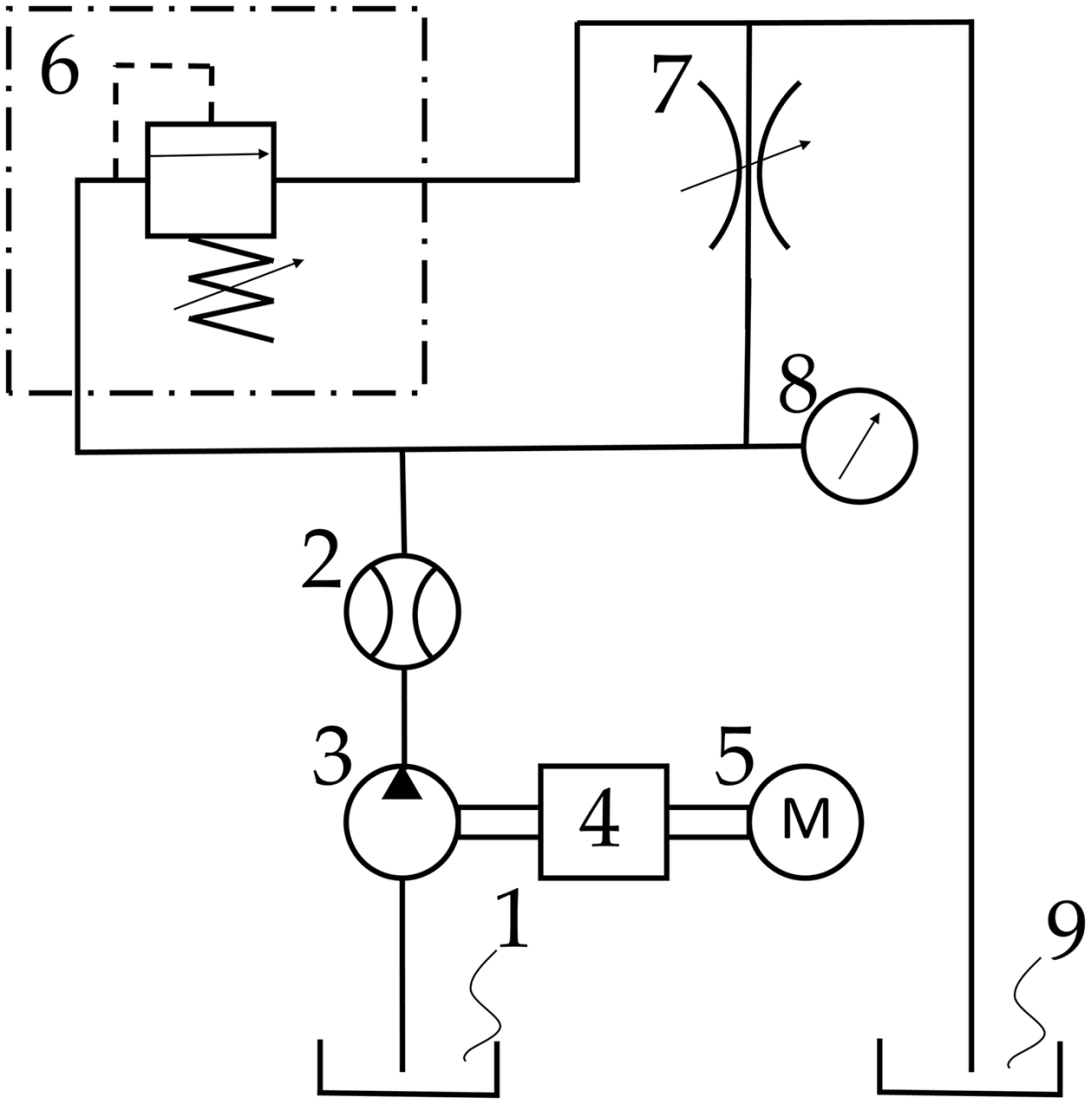
Diagram of the measurement setup, where: 1 – oil tank on the suction side, 2 – flowmeter, 3 – gear pump, 4 – torque meter with rotational speed measurement function, 5 – electric motor, 6 – relief valve, 7 – throttle valve, 8 – pressure gauge, 9 – oil tank on the discharge side.

The pump was installed in a system as shown in Fig 1, where the load on the pump was regulated using a throttle valve within a range of 0 to 20 MPa, while the pump operated at a set initial rotational speed of approximately 2000 rpm.

The measurement accuracy characteristics of the devices used during the experiment are presented in Table 2.

**Table 2:** Measurement accuracy of the devices used in the experiment.

Torque meter with rotational speed measurement function	
Torque measurement range:	up to 15 Nm
Torque measurement accuracy:	±0.5%
Torque measurement resolution:	0.01 Nm
Rotational speed range:	0–5000 rpm
Rotational speed accuracy:	±1%
Rotational speed resolution:	0.1 rpm
Flow meter:	
Flow measurement range:	0.1–6 l/min (typical range for oils with viscosity of 10–1000 mm²/s)
Flow measurement accuracy:	±0.3%
Flow measurement resolution:	0.1 l/min
Pressure gauge:	
Pressure measurement range:	0–25 MPa
Accuracy class:	1.6
Resolution:	0.1 MPa

Gear pumps used in this study are external gear-type pumps consisting of two mating spur gears enclosed within a close-fitting housing. Each gear rotates on its own axis, with one being driven and the other idling. The meshing of the gear teeth and their rotation causes the trapping and transport of fluid from the inlet to the outlet side of the pump.

While these devices appear mechanically simple, their performance is significantly influenced by the geometry and tolerance of the internal components, particularly the overflow paths—very narrow clearances between the gear profiles and the surrounding housing (radial and axial leakage gaps). These internal gaps affect:

- Volumetric efficiency – too large clearance leads to increased internal leakage,
- Hydrodynamic friction – excessively tight fits can increase friction and thermal expansion risk,
- Cavitation risk – improperly vented or sealed gaps can cause vapor bubble formation.

The most critical leakage paths are:

- Radial clearance: between the external gear diameter and the inner contour of the pump housing,
- Axial clearance: between the gear face (tooth width) and the pump side walls,
- Tooth intermesh clearance: dependent on profile correction and backlash.

The pump used in this study features gears with a module of 2 mm, 9 teeth, and a corrected involute profile (x=+0.4x = +0.4x=+0.4) to prevent undercutting. The external diameter of the gear is approximately 23.6 mm, with a gear width of 4.00 mm. Precision fits were applied in both radial and axial directions to control internal leakage. The Table 3 below summarizes the tolerances used in these regions.

**Table 3:** Mechanical fits for gear-to-housing interfaces in the test pump.

Interface	Nominal Dimension	Fit Type	Gear Range (mm)	Housing Range (mm)	Radial/Axial Clearance
External gear diameter	23.6 mm	H8/f8	23.508–23.557	23.600–23.633	0.033–0.125 mm
Gear face width	4.00 mm	H7/h6	3.992–4.000	4.000–4.015	0.000–0.023 mm

These precise clearances ensure the pump operates with minimal backflow while maintaining the necessary thermal tolerance and lubrication film thickness. The magnitude of internal leakage through these overflow paths is also a key consideration in interpreting the dynamic response of the pump, particularly in transient operating regimes.

**Fig 2:**
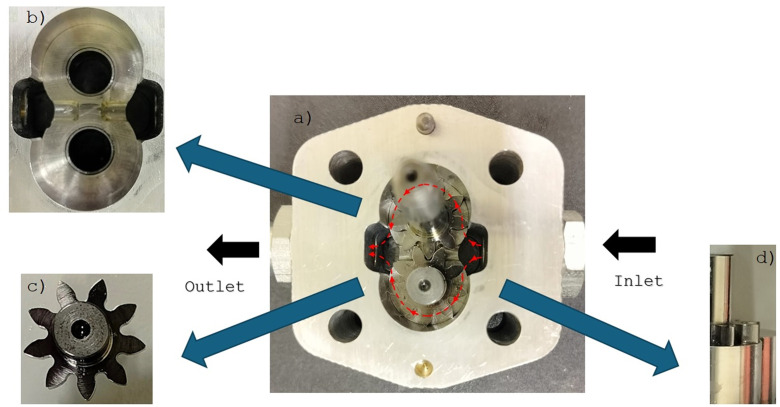
Actual view of the inside of a gear pump symbol 1.40.09.00.105 manufactured by Agricola Hydraulika Siłowa Sp. z o. o. Sp. k. from (Lubień Kujawski, Poland): a) view of the pump after splitting, b) view of the bearing housing with the pumping chamber, c) view of the gear wheel, d) vertical view of the gear wheel with the bearing housing.

Fig 2 shows the construction of the pump. The inlet diameter is 8.83 mm, and the outlet diameter is 8.53 mm.

The study utilized oils commonly used in machinery and vehicles, primarily intended for engines, gear systems, hydraulic systems, shock absorbers, air conditioning systems, chain saw lubrication, and two-stroke spark-ignition engines lubricated by a fuel-oil mixture. The characteristics of the oils are presented in Table 4. The oils were previously analyzed with respect to their kinematic viscosity at 25°C and 50°C [35].

**Table 4:** Characteristics of the Tested Oils.

Sample no.	Kinematic viscosity at 25 °C [35]	Kinematic viscosity at 50 °C [35]	Application	SAE Viscosity Grade or Other Designations	Series	Manufacturer	City, Country
1	65.02	28.00	Combustion engine oil	5W/20	Energy Ultra JP	Mannol (SCT-Vertriebs GmbH)	Wedel, Niemcy
2	99.39	39.59	Combustion engine oil	0W/30	Legend Extra	Mannol (SCT-Vertriebs GmbH)	Wedel, Niemcy
3	229.82	70.32	Combustion engine oil	15W/40	Universal	Mannol (SCT-Vertriebs GmbH)	Wedel, Niemcy
4	280.50	85.98	Combustion engine oil	20W/50	Lubro	Orlen Oil (Orlen Group)	Trzebinia, Polska
5	320.75	115.37	Combustion engine oil	10W/60	Synthoil Race Tech GT1	Liqui Moly	Ulm, Niemcy
6	58.73	22.20	Hydraulic oil	L-HL 32	Hydrol	Orlen Oil (Orlen Group)	Trzebinia, Polska
7	87.79	28.55	Hydraulic oil	HL 46	Revline	Flukar sp. z o.o.	Katowice, Polska
8	118.65	43.76	Hydraulic oil (for air conditioning systems)	PAO 68	Hart	Airstal sp. z o o.	Koluszki, Polska
9	21.33	10.85	Shock absorber oil	15-WL 150	Amortyzol	Orlen Oil (Orlen Group)	Trzebinia, Polska
10	269.68	82.17	Gear oil	GL-4 80W/90	Hipol	Orlen Oil (Orlen Group)	Trzebinia, Polska
11	295.70	81.26	Gear oil	GL-5 85W/90	Hipol 15F	Orlen Oil (Orlen Group)	Trzebinia, Polska
12	784.50	171.05	Gear oil	85W/140 GL-5 LS	Hypoid LSD	Mannol (SCT-Vertriebs GmbH)	Wedel, Niemcy
13	61.09	25.54	Combustion engine oil for hybrid vehicles	0W/16	Hybrid SP	Mannol (SCT-Vertriebs GmbH)	Wedel, Niemcy
14	126.16	39.68	Chainsaw lubrication oil	VG 68	Pilarol	Orlen Oil (Orlen Group)	Trzebinia, Polska
15	54.63	21.34	For two-stroke spark-ignition engines with a mixture lubrication system	0W/12	Mixol S	Orlen Oil (Orlen Group)	Trzebinia, Polska
16	180.07	55.18	For two-stroke spark-ignition engines with a mixture lubrication system	0W/20	2T Semisythetic	Orlen Oil (Orlen Group)	Trzebinia, Polska

The tests were conducted at an ambient temperature of approximately 24 ± 1°C. Oils were pumped from the tank on the suction side to the tank on the discharge side to prevent heating by the pump in a closed circuit. This setup allowed for the maintenance of realistic operating conditions for the pump during oil transport. The experiments were performed for five operating states resulting from the load generated by the throttle valve, ranging from 0 to 20 MPa in intervals of 5 MPa.

**Fig 3:**
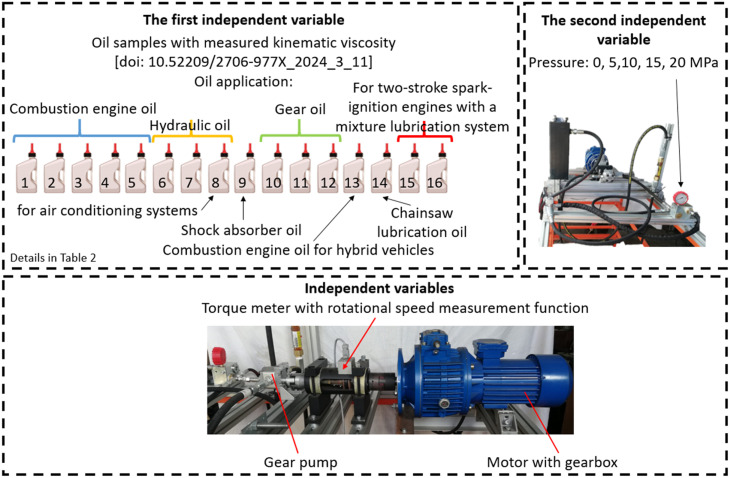
Dependent and independent variables in the experimental study.

The experimental program involved the investigation of two independent variables: the type of oil (16 variants) and the system pressure during oil pumping (ranging from 0 to 20 MPa, in 5 MPa increments). The dependent variables measured were the torque and rotational speed at the input shaft of the gear pump. The elements of the experimental design are presented in Fig 3.

In the analysis of the measurement error, the arithmetic mean was taken as the estimator of the desired value, for which the confidence interval was calculated for the confidence interval p = 0.05. Statistical analysis was performed in accordance with the procedures appropriate for the normal distribution of the measured measurement points.

## 3. Results and discussion

The results of the study include measurements of torque *T*, rotational speed *n*, and mechanical power *P* on the input shaft of the gear pump, depending on the type of oil (no. 1–16) and the pressure at which the pump operated. These results are presented in Table 5.

**Table 5:** Results of the measurements of torque T, rotational speed n, and mechanical power P on the input shaft of the gear pump depending on the type of oil (no. 1–16) and pumping pressure, where: avg – average value,p = 0.05 – the confidence interval.

Oil No. 1										
**Pressure**	**0 MPa**		**5 MPa**		**10 MPa**		**15 MPa**		**20 MPa**	
	avg	p = 0.05	avg	p = 0.05	avg	p = 0.05	avg	p = 0.05	avg	p = 0.05
T, Nm	0.18	0.0015	0.49	0.0029	1.39	0.0048	2.41	0.0098	3	0.0027
n, rpm	1928.5	0.0520	1918	0.0240	1906.5	0.0706	1892.9	0.1406	1882	0.0415
P, W	35.7	0.3178	98.4	0.5766	277.9	0.9408	476.9	1.9326	592.2	0.5406
**Oil No. 2**										
**Pressure**	**0 MPa**		**5 MPa**		**10 MPa**		**15 MPa**		**20 MPa**	
	avg	p = 0.05	avg	p = 0.05	avg	p = 0.05	avg	p = 0.05	avg	p = 0.05
T, Nm	0.15	0.0052	0.58	0.0025	1.48	0.0034	3.25	0.0023	0.17	0.0015
n, rpm	1934	0.0111	1921.9	0.0218	1909.6	0.0588	1884	0.0411	1928.5	0.0520
P, W	30.8	1.0613	116.8	0.5168	296.2	0.6909	642.7	0.4610	35.7	0.3178
**Oil No. 3**										
**Pressure**	**0 MPa**		**5 MPa**		**10 MPa**		**15 MPa**		**20 MPa**	
	avg	p = 0.05	avg	p = 0.05	avg	p = 0.05	avg	p = 0.05	avg	p = 0.05
T, Nm	0.10	0.0010	0.70	0.0031	1.64	0.0033	2.41	0.0041	3.34	0.0041
n, rpm	1942.5	0.0384	1927.1	0.0969	1912.5	0.0550	1900.4	0.0717	1885	0.0597
P, W	22.1	0.2019	141.7	0.6396	327.8	0.6608	480.8	0.8083	660.9	0.8182
**Oil No. 4**										
**Pressure**	**0 MPa**		**5 MPa**		**10 MPa**		**15 MPa**		**20 MPa**	
	avg	p = 0.05	avg	p = 0.05	avg	p = 0.05	avg	p = 0.05	avg	p = 0.05
T, Nm	0.10	0.0042	0.72	0.0038	1.59	0.0033	2.64	0.0056	3.34	0.0020
n, rpm	1928.2	0.0264	1916.1	0.0353	1905	0.0660	1890.5	0.0750	1881	0.0423
P, W	21.1	0.8492	144.7	0.7784	317.6	0.6669	524.3	1.1178	658.1	0.4067
**Oil No. 5**										
**Pressure**	**0 MPa**		**5 MPa**		**10 MPa**		**15 MPa**		**20 MPa**	
	avg	p = 0.05	avg	p = 0.05	avg	p = 0.05	avg	p = 0.05	avg	p = 0.05
T, Nm	0.11	0.0008	0.71	0.0017	1.59	0.0067	2.76	0.0075	3.61	0.0026
n, rpm	1926.2	0.0008	1914.8	0.0553	1904.2	0.0671	1888.9	0.0950	1876.4	0.0371
P, W	22.4	0.0008	143.1	0.3542	318.1	1.3490	547	1.4702	710.3	0.5300
**Oil No. 6**										
**Pressure**	**0 MPa**		**5 MPa**		**10 MPa**		**15 MPa**		**20 MPa**	
	avg	p = 0.05	avg	p = 0.05	avg	p = 0.05	avg	p = 0.05	avg	p = 0.05
T, Nm	0.11	0.0071	0.57	0.0026	1.53	0.0029	2.36	0.0076	3.53	0.0019
n, rpm	1930	0.0087	1919.9	0.0335	1907.9	0.0339	1896.4	0.1600	1881.2	0.0396
P, W	22.4	1.4568	114.8	0.5298	307.5	0.5868	469.2	1.5002	697	0.3770
**Oil No. 7**										
**Pressure**	**0 MPa**		**5 MPa**		**10 MPa**		**15 MPa**		**20 MPa**	
	avg	p = 0.05	avg	p = 0.05	avg	p = 0.05	avg	p = 0.05	avg	p = 0.05
T, Nm	0.16	0.0009	0.50	0.0013	1.34	0.0036	2.19	0.0106	3.06	0.0031
n, rpm	1959.8	0.0342	1936.7	0.0435	1917.9	0.0528	1904.2	0.1374	1889.5	0.0408
P, W	32.8	0.1842	102.1	0.2740	270.3	0.7223	437.4	2.0868	606.2	0.6088
**Oil No. 8**										
**Pressure**	**0 MPa**		**5 MPa**		**10 MPa**		**15 MPa**		**20 MPa**	
	avg	p = 0.05	avg	p = 0.05	avg	p = 0.05	avg	p = 0.05	avg	p = 0.05
T, Nm	0.07	0.0006	0.74	0.0029	1.52	0.0023	2.41	0.0052	3.50	0.0015
n, rpm	1928.6	0.0389	1917.7	0.0458	1906.5	0.0489	1895.4	0.0894	1880.2	0.0268
P, W	13.9	0.1297	148.8	0.5906	305	0.4627	478.5	1.0235	690.9	0.2987
**Oil No. 9**										
**Pressure**	**0 MPa**		**5 MPa**		**10 MPa**		**15 MPa**		**20 MPa**	
	avg	p = 0.05	avg	p = 0.05	avg	p = 0.05	avg	p = 0.05	avg	p = 0.05
T, Nm	0.13	0.0010	0.53	0.0016	1.39	0.0030	2.25	0.0047	3.48	0.0029
n, rpm	1930	0.0109	1920.2	0.0301	1908.9	0.0608	1897.7	0.0656	1881.2	0.0428
P, W	27.5	0.2190	106.6	0.3317	278.8	0.6028	448.4	0.9228	686.7	0.5597
**Oil No. 10**										
**Pressure**	**0 MPa**		**5 MPa**		**10 MPa**		**15 MPa**		**20 MPa**	
	avg	p = 0.05	avg	p = 0.05	avg	p = 0.05	avg	p = 0.05	avg	p = 0.05
T, Nm	0.01	0.0051	0.86	0.0024	1.83	0.0018	2.63	0.0039	3.48	0.0036
n, rpm	1948.4	0.0390	1933.8	0.0311	1917	0.0426	1904.3	0.0616	1890	0.0244
P, W	2.2	1.0440	175.1	0.4888	368.5	0.3752	525.3	0.7762	688.9	0.7084
**Oil No. 11**										
**Pressure**	**0 MPa**		**5 MPa**		**10 MPa**		**15 MPa**		**20 MPa**	
	avg	p = 0.05	avg	p = 0.05	avg	p = 0.05	avg	p = 0.05	avg	p = 0.05
T, Nm	0.03	0.0038	0.80	0.0011	1.78	0.0056	2.83	0.0020	3.54	0.0020
n, rpm	1930.1	0.0223	1918	0.0125	1905.5	0.0674	1891.4	0.0350	1880.3	0.0275
P, W	5.3	0.7720	161.9	0.2255	356.3	1.1327	561.6	0.4008	697.3	0.3946
**Oil No. 12**										
**Pressure**	**0 MPa**		**5 MPa**		**10 MPa**		**15 MPa**		**20 MPa**	
	avg	p = 0.05	avg	p = 0.05	avg	p = 0.05	avg	p = 0.05	avg	p = 0.05
T, Nm	0.01	0.0006	0.89	0.0027	1.85	0.0017	2.82	0.0061	3.73	0.0040
n, rpm	1926	0.0044	1914.8	0.0414	1902.6	0.0389	1889.4	0.0789	1876.4	0.0444
P, W	1.9	0.1278	177.5	0.5465	370.3	0.3507	558.1	1.2055	734.7	0.7823
**Oil No. 13**										
**Pressure**	**0 MPa**		**5 MPa**		**10 MPa**		**15 MPa**		**20 MPa**	
	avg	p = 0.05	avg	p = 0.05	avg	p = 0.05	avg	p = 0.05	avg	p = 0.05
T, Nm	0.09	0.0048	0.57	0.0010	1.53	0.0026	2.42	0.0041	3.44	0.0012
n, rpm	1931.9	0.0188	1921.9	0.0216	1909.2	0.0310	1896.6	0.1178	1882.7	0.0335
P, W	18.8	0.9795	115.3	0.2205	306.3	0.5193	480.7	0.8250	679	0.2462
**Oil No. 14**										
**Pressure**	**0 MPa**		**5 MPa**		**10 MPa**		**15 MPa**		**20 MPa**	
	avg	p = 0.05	avg	p = 0.05	avg	p = 0.05	avg	p = 0.05	avg	p = 0.05
T, Nm	0.07	0.0007	0.63	0.0011	1.61	0.001	2.50	0.0040	3.47	0.0012
n, rpm	1929.2	0.0409	1918.3	0.0306	1906.7	0.0387	1895.2	0.0584	1881.6	0.0301
P, W	13.7	0.1350	126.1	0.2318	322.2	0.2839	497.5	0.7860	682.8	0.2527
**Oil No. 15**										
**Pressure**	**0 MPa**		**5 MPa**		**10 MPa**		**15 MPa**		**20 MPa**	
	avg	p = 0.05	avg	p = 0.05	avg	p = 0.05	avg	p = 0.05	avg	p = 0.05
T, Nm	0.12	0.0008	0.64	0.0020	1.56	0.0012	2.40	0.0038	3.43	0.0016
n, rpm	1928.3	0.0339	1917.9	0.0196	1906.7	0.0387	1895.7	0.0572	1882.6	0.0383
P, W	25.2	0.1695	130.1	0.4086	311.8	0.2583	478.1	0.7591	677.6	0.3278
**Oil No. 16**										
**Pressure**	**0 MPa**		**5 MPa**		**10 MPa**		**15 MPa**		**20 MPa**	
	avg	p = 0.05	avg	p = 0.05	avg	p = 0.05	avg	p = 0.05	avg	p = 0.05
T, Nm	0.09	0.0011	0.75	0.0039	1.68	0.0036	2.65	0.0061	3.55	0.0020
n, rpm	1928	0.0529	1915.8	0.0431	1903.8	0.0513	1890.8	0.0698	1878	0.0085
P, W	18.5	0.2328	151.8	0.7961	336.5	0.7233	526.2	1.2150	699.7	0.3948

**Fig 4:**
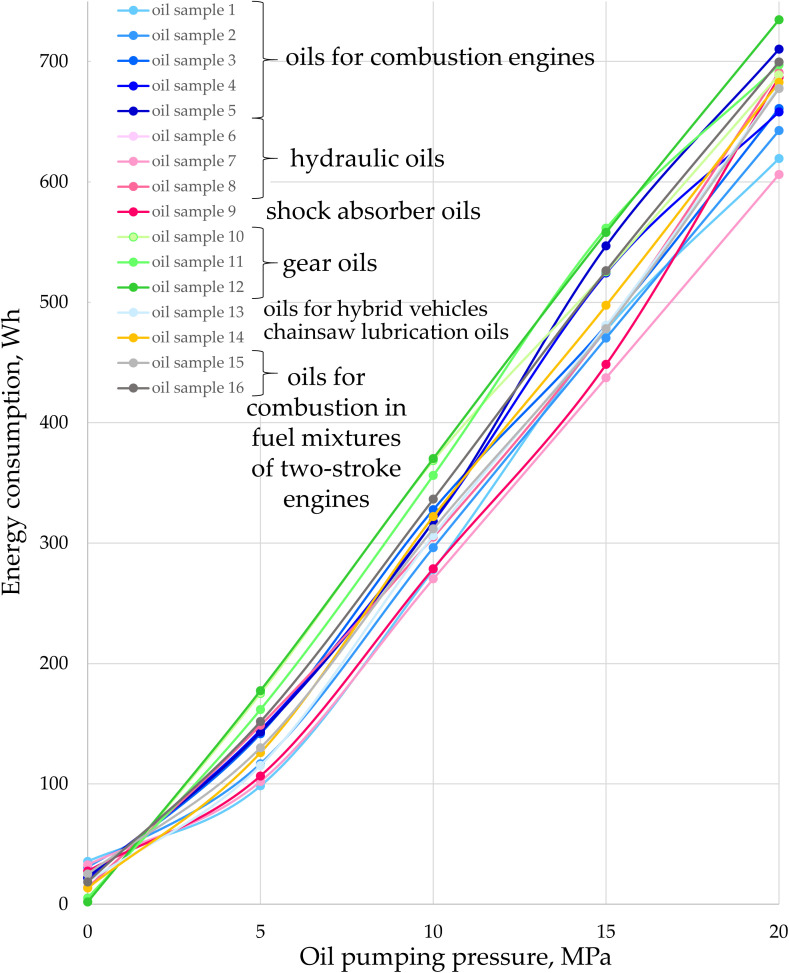
Energy consumption at the input shaft of the gear pump depending on the pumped oil pressure and type of oil, at the recommended pump speed for continuous operation (2000 rpm).

The impact of pressure and type of oil on the energy demand of a gear pump is illustrated in Fig 4. It can be observed that energy consumption increases with the rise in oil pressure, consistent with the findings of Hargreaves and Planitz in 2009 [36]. For idle operation of the gear pump, i.e., at a pressure close to 0 MPa, energy consumption ranges from 5 to 35 Wh. As pressure increases, regardless of the type of oil, energy consumption rises accordingly: for 5 MPa, it ranges from 98 to 175 Wh; for 10 MPa, from 307 to 368 Wh; for 15 MPa, from 437 to 525 Wh; and for 20 MPa, from 606 to 734 Wh. The highest energy consumption is observed for gear oils (green curves in Fig 4), while hydraulic oils and engine oils exhibit the lowest consumption. The percentage increase in energy consumption between the least and most energy-intensive oils depends on the pressure during pumping and is approximately 600% at 0 MPa, 78% at 5 MPa, 20% at 10 MPa, 20% at 15 MPa, and 21% at 20 MPa. It is notable that at higher pressures (from 10 MPa to 20 MPa), particularly at the recommended maximum operating pressure of 200 MPa (which corresponds to optimal pump efficiency within the recommended working range), the difference in energy consumption due to the type of oil is around 20%. Furthermore, during pump operation with the oil recommended for hydraulic systems (test no. 7), energy consumption is lowest at the recommended and most efficient operating conditions (2000 rpm and 20 MPa).

**Fig 5:**
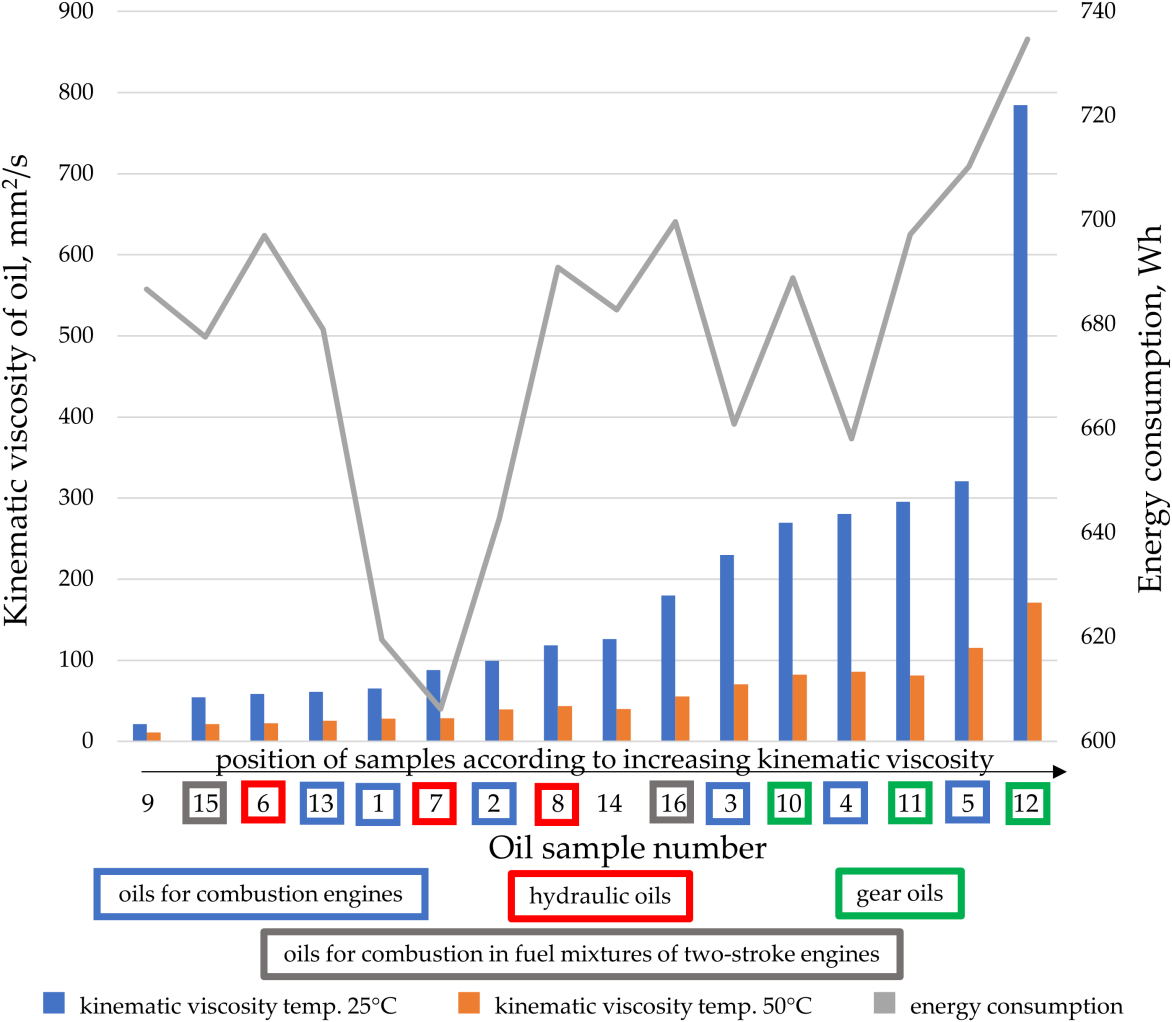
The kinematic viscosity values for the tested oil samples at temperatures of 25°C and 50°C, as well as the energy consumption during oil transfer at a pressure of 20 MPa and a gear pump speed of 2000 rpm.

**Fig 6:**
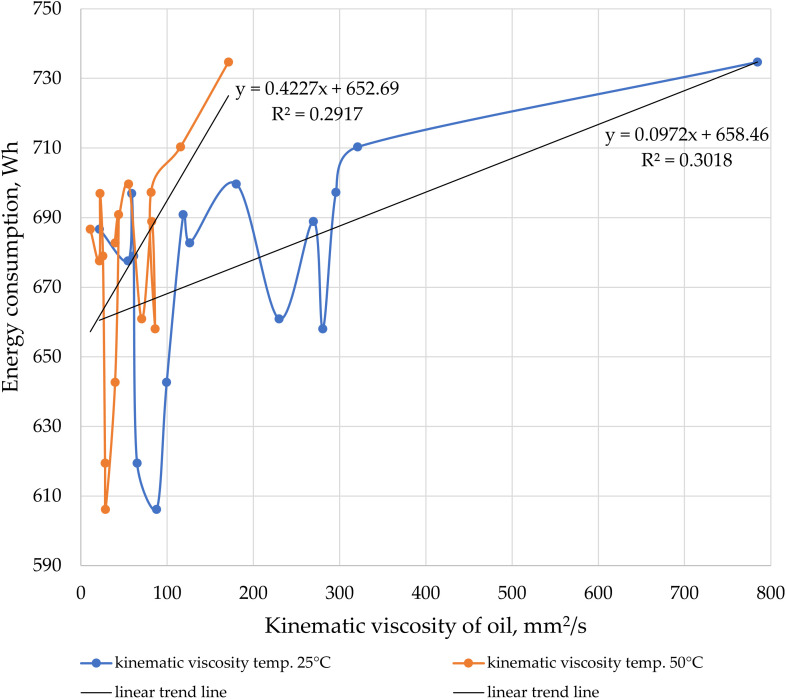
Energy consumption of the gear pump at a rotational speed of 2000 rpm and a pressure of 20 MPa during the transfer of oils with varying kinematic viscosities at temperatures of 25°C and 50°C.

The kinematic viscosity of oil affects the energy consumption of a gear pump during fluid transfer, as viscosity directly influences the flow resistance within the hydraulic system. Higher oil viscosity means the fluid offers greater resistance to movement, leading to increased frictional losses and flow resistance [37]. The higher the kinematic viscosity, the more difficult it is for the pump to transfer oil through the lines and other components of the system. Oil with higher viscosity creates greater flow resistance, forcing the pump to perform more work to move the fluid [38]. In the case of a gear pump, higher viscosity results in increased energy losses due to internal friction between the pump gears and the oil [39]. As a result, part of the energy supplied by the pump is lost as heat, rather than being effectively used to transfer the oil [40]. The graph shown in Fig 5 indicates that oils used in machinery and vehicles may exhibit different behavior compared to conventional oils due to various additives. A comparison of oils available on the market for machinery and vehicles does not show a consistent relationship between increased energy consumption by the pump and the pumping of oils with higher kinematic viscosity (for the recommended pump operation at its most efficient speed of 2000 rpm and pressure of 20 MPa). The viscosity characteristics at 25°C and 50°C, and energy consumption charts (Fig 6), deviate significantly from linear or other simple functions describing this phenomenon. There are several reasons why not all oils lead to an increase in energy consumption by the gear pump with rising kinematic viscosity. Different physical and chemical properties of oils can affect how they behave in the hydraulic system. Some oils contain special additives, such as viscosity modifiers, which improve their flow properties depending on temperature [41,42]. These additives allow the oil to perform better across a wide range of temperatures without causing excessive increases in viscosity or system resistance. Consequently, even with rising kinematic viscosity, flow resistance does not increase significantly, which limits the pump’s energy consumption. Sometimes, an increase in kinematic viscosity may result from other physical properties, such as increased oil density, rather than a significant rise in dynamic viscosity. If the dynamic viscosity does not change drastically, the flow resistance and energy consumption may remain stable. Kinematic viscosity is the ratio of dynamic viscosity to fluid density, and different oils have different molecular structures, which influence how the fluid behaves during flow [43]. Oils with a low internal friction coefficient may flow more efficiently even at higher kinematic viscosity, reducing the pump’s load and minimizing energy consumption. Some oils may exhibit non-Newtonian behavior, meaning their viscosity changes depending on flow velocity or shear stress [44,45]. Such oils can show lower flow resistance at high speeds or under heavy loads, reducing the pump’s energy consumption despite a higher kinematic viscosity at rest. In some systems, hydraulic pumps may operate less efficiently at very low viscosities, as the oil may not provide sufficient lubrication and sealing [46,47]. In such cases, increasing viscosity can improve system efficiency by providing better lubrication and reducing internal leakage. As a result, higher viscosity can reduce energy consumption instead of increasing it. Energy consumption by the pump can also be influenced by cavitation, especially when pumping higher-viscosity oils [48]. Some gear pumps are designed to work efficiently with higher-viscosity oils. The pump’s construction, gear geometry, and clearances between components may be optimized for a specific viscosity range, allowing efficient fluid transfer without excessive energy consumption [49–51]. This phenomenon is well illustrated in Fig 5, where the pump is specifically designed to pump oil number 7 (hydraulic oil HL46). Despite not being the lowest viscosity oil, the transfer of this oil is associated with the lowest energy consumption.

**Fig 7:**
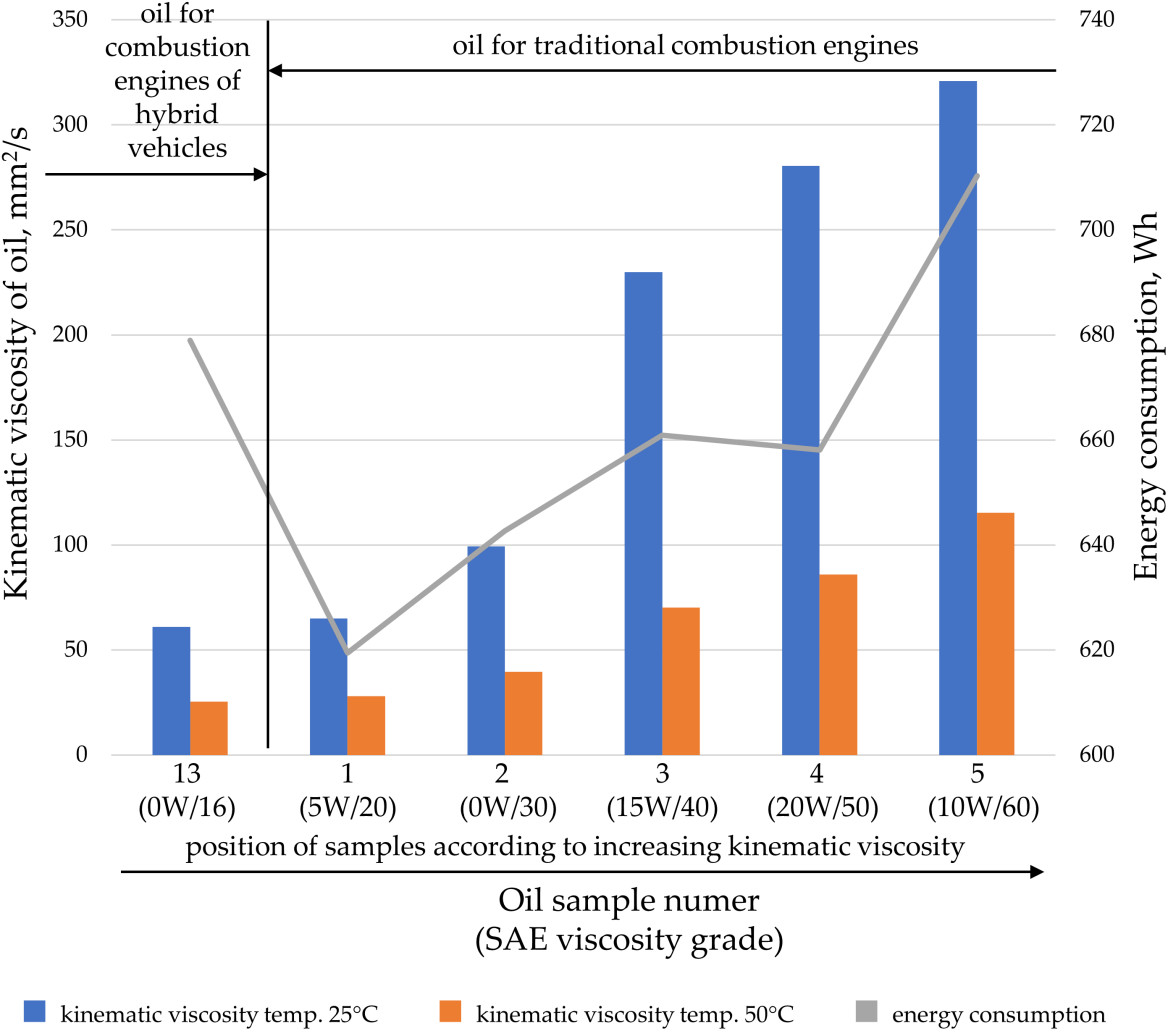
The kinematic viscosity values for the tested oil samples intended for internal combustion engines at temperatures of 25°C and 50°C, as well as the energy consumption during oil transfer at a pressure of 20 MPa and a gear pump speed of 2000 rpm.

**Fig 8:**
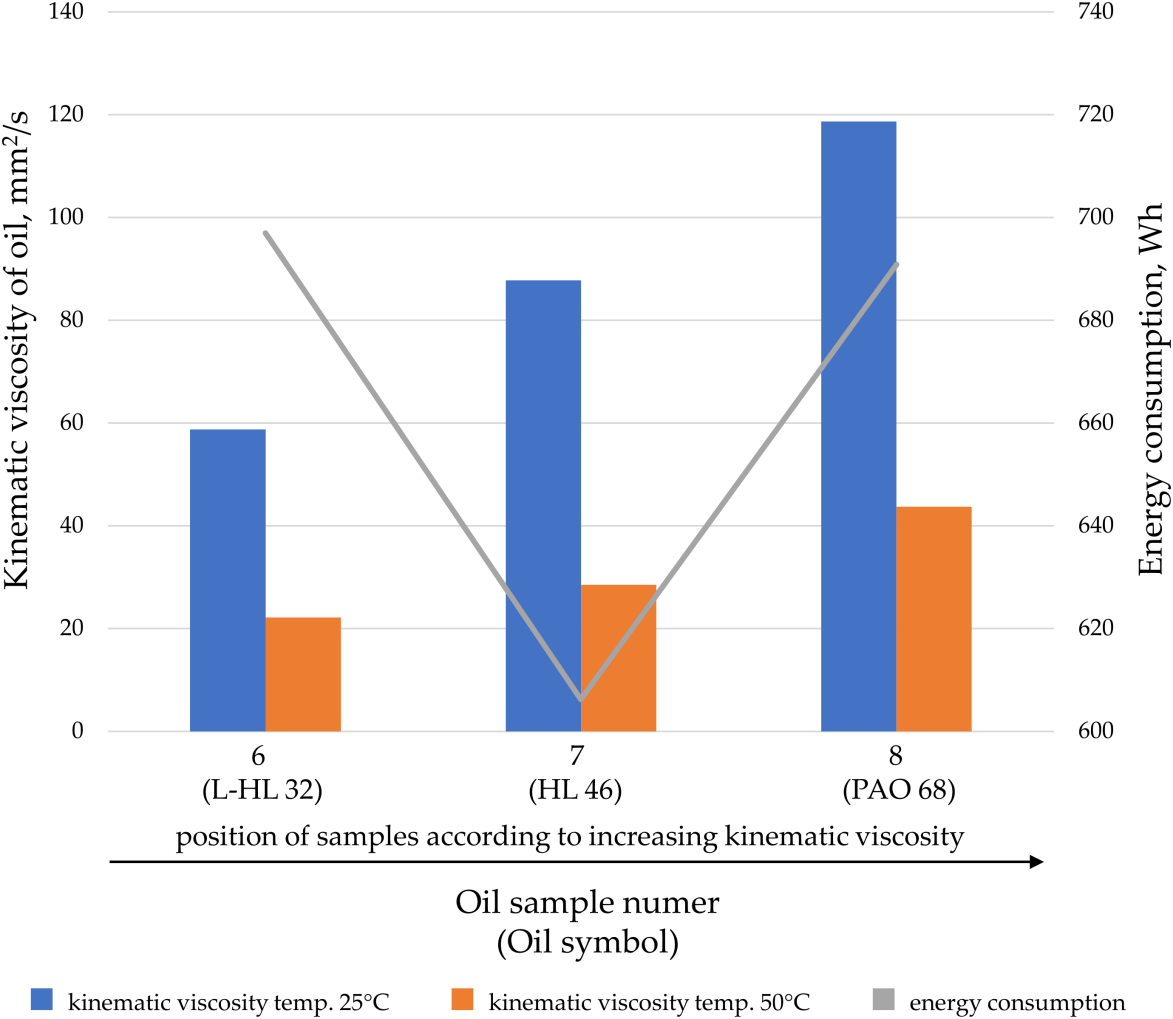
The kinematic viscosity values for the tested hydraulic oil samples at temperatures of 25°C and 50°C, as well as the energy consumption during oil transfer at a pressure of 20 MPa and a gear pump speed of 2000 rpm.

**Fig 9:**
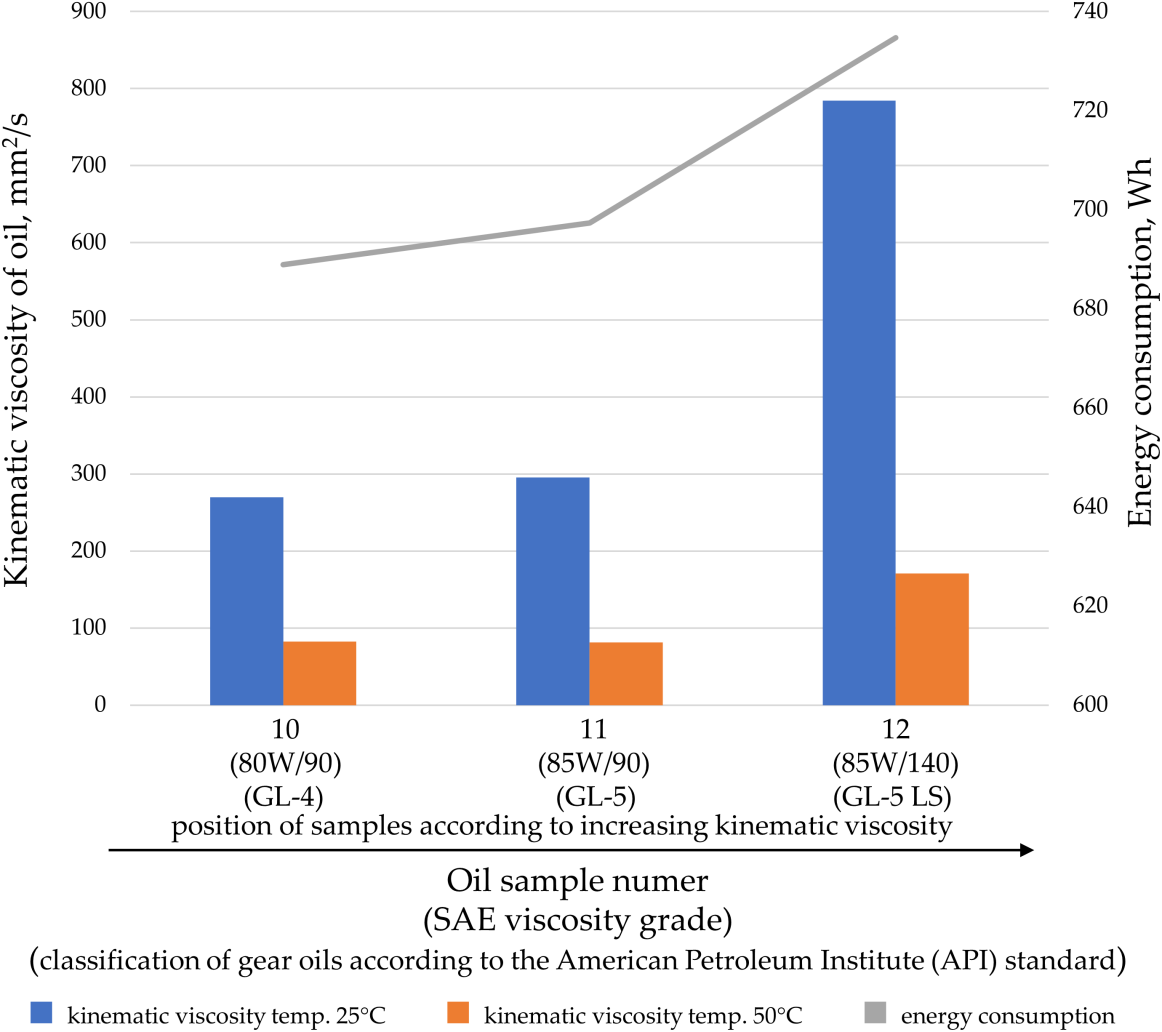
The kinematic viscosity values for the tested gear oil samples at temperatures of 25°C and 50°C, as well as the energy consumption during oil transfer at a pressure of 20 MPa and a gear pump speed of 2000 rpm.

**Fig 10:**
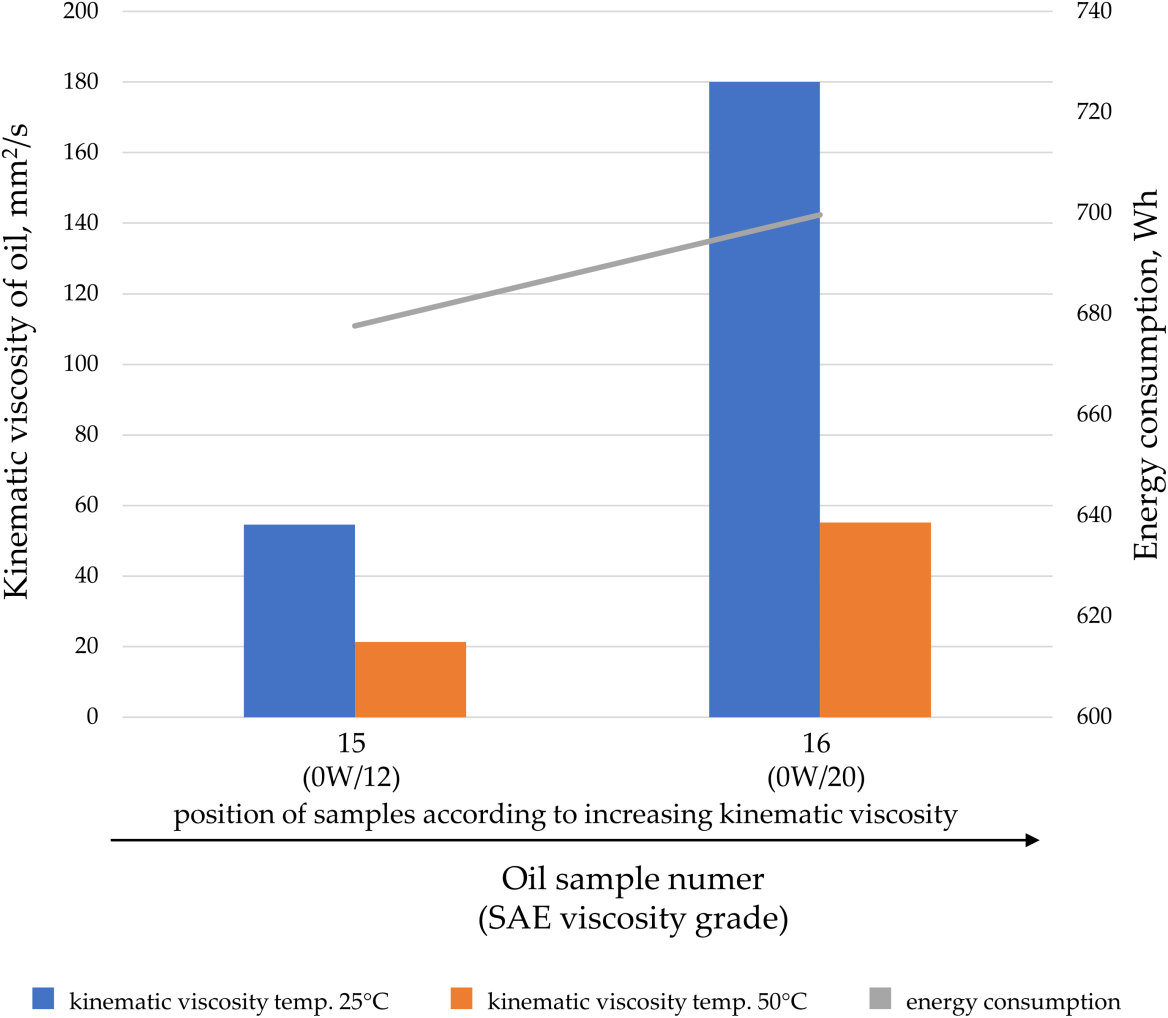
The kinematic viscosity values for the tested oil samples intended for two-stroke engine fuel mixtures at temperatures of 25°C and 50°C, as well as the energy consumption during oil transfer at a pressure of 20 MPa and a gear pump speed of 2000 rpm.

Oils can be characterized based on their kinematic or dynamic viscosity, but they can also be categorized according to their application. Oils within a specific application group may feature special additives that alter their properties during transfer. Analyses were conducted on the impact of kinematic viscosity on energy consumption, divided by oil application: engine oil (Fig 7), hydraulic oil (Fig 8), gear oils (Fig 9), and oils for combustion in fuel mixtures intended for two-stroke engines (Fig 10).

Engine oils for traditional internal combustion engines show a higher energy demand from the gear pump as the oil’s kinematic viscosity increases (Fig 7, oil samples 1–5). These values also rise in accordance with the SAE (Society of Automotive Engineers) classification for summer oils, or those without the “W” designation. The group of tested engine oils also included one designed for hybrid vehicles (Fig 7, oil sample 13), which had the lowest kinematic viscosity and a summer SAE designation. However, it did not exhibit the lowest energy consumption during pumping, which deviates from general trends. This may be due to the fact that engine oils for hybrid vehicles are designed to ensure maximum fuel efficiency and lubrication from the first seconds after engine start-up [52,53]. In hybrid vehicles, frequent engine restarts occur, and the oil must protect against harmful phenomena such as knocking combustion. Using low-viscosity oils is advantageous in hybrid engines, as they minimize friction, contributing to fuel savings and reducing electrical energy consumption during start-up. Oil in hybrid vehicles differs from oil for purely internal combustion vehicles, as it must handle frequent engine shutdowns, lower operating temperatures, and rapid lubrication after start-up. Therefore, hybrid vehicle oils often have lower viscosity, contain more cleaning and anti-corrosion additives, and exhibit better resistance to contamination. In contrast, oils for traditional internal combustion engines are more suited for prolonged operation at higher temperatures and heavier loads, which explains their higher viscosity and different technical characteristics.

In internal combustion engines, friction modifiers [54,55] are used to reduce friction between moving engine parts, minimizing energy losses due to friction. These modifiers contain organic compounds such as fatty acid esters or graphite, which form a thin layer on metal surfaces, reducing resistance. Lower friction allows for more efficient fuel utilization, leading to fuel savings and reduced exhaust emissions. Viscosity index improvers adjust the oil’s viscosity characteristics depending on temperature, providing better viscosity stability across a wide temperature range [56,57]. These polymers “stretch” at higher temperatures to prevent excessive viscosity drop and “contract” at lower temperatures to maintain oil fluidity. This ensures optimal viscosity in both low and high temperatures, reducing start-up friction and providing more effective lubrication under heavy loads. Anti-wear additives create a thin protective layer on metal surfaces, reducing wear and friction between moving parts [58]. Zinc dialkyldithiophosphate (ZDDP) is a commonly used compound that acts as a protective buffer, especially in areas of high pressure, preventing component wear. These additives reduce engine friction, prolong component lifespan, and consequently lower energy consumption. Dispersant and detergent additives maintain the cleanliness of internal engine parts, preventing the formation of deposits and varnish [59]. Detergents, such as calcium sulfonates, prevent deposit build-up, while dispersants suspend contaminants in the oil, preventing them from settling. Fewer deposits and contaminants in the engine allow the oil to circulate smoothly, reducing energy losses. Pour point depressants enable oil to remain fluid at very low temperatures, preventing freezing [60]. These polymer compounds inhibit the formation of paraffin crystals in the oil under cold conditions, facilitating easier engine start-up in low temperatures and reducing the energy needed to start the vehicle on cold days. Antioxidants slow the oxidation processes in oil that lead to its degradation and the formation of harmful acids and deposits [61]. The most commonly used antioxidants are amine and phenolic inhibitors, which help maintain the oil in optimal condition for longer periods, reducing the need for frequent changes and improving energy efficiency. Extreme Pressure (EP) additives provide additional protection under extreme load conditions when standard additives may not suffice [62]. Sulfides, phosphates, and chlorine compounds activate at high temperatures, forming a protective coating on metal surfaces. These additives reduce component wear and minimize friction under heavy loads, improving energy efficiency. Lubricity enhancers reduce friction between moving engine components, enhancing the lubricating properties of the oil [63]. Organic esters or polymers strengthen the oil’s natural lubricating qualities, reducing fuel consumption and improving engine efficiency, especially in modern high-power engines.

Hydraulic oils, with increasing kinematic viscosity, are expected to require higher energy consumption by the gear pump. As shown in Fig 8, this trend is evident for samples 6 and 8, which are the hydraulic oils with the lowest and highest kinematic viscosities. However, the hydraulic oil with an intermediate viscosity shows significantly lower energy consumption during transfer (the lowest among the tested oils). This phenomenon is likely due to the previously described relationship, stemming from the pump’s design, which is optimized for the viscosity and characteristics of HL 46 oil (sample 7). This is the oil recommended by the gear pump manufacturer for hydraulic systems. When designing such pumps, manufacturers often aim for the lowest possible energy consumption under nominal or recommended operating conditions.

Gear oils (Fig 9) and oils intended for fuel mixtures (Fig 10) exhibit a predictable increase in energy consumption as the kinematic viscosity increases.

**Fig 11:**
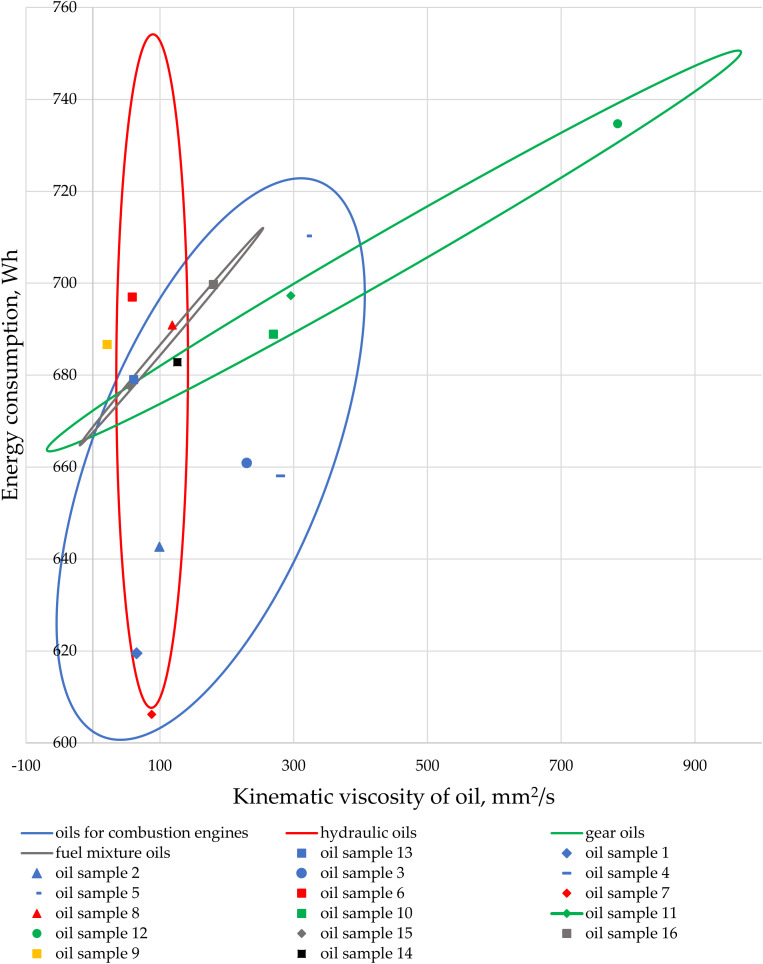
The comparison of energy consumption for pumping oils intended for machinery and vehicles using a gear pump, with distinct areas (ellipses) highlighted based on oil application, is presented.

The comparison of energy consumption for pumping oils intended for machinery and vehicles using a gear pump, categorized by oil applications, is presented in Fig 11. The ellipses in Fig 11 highlight the variability in energy demand during the transfer of different types of oils.

A multi-criteria statistical significance analysis was also performed. A multifactorial ANOVA was conducted to examine the effect of two factors: kinematic viscosity and oil application, on energy consumption. The ANOVA results are presented in Table 6. The dependent variable in the analysis is energy consumption, while the two independent variables are kinematic viscosity (continuous variable) and application (categorical variable). The analysis was based on a linear model, where energy consumption depends on both kinematic viscosity and oil application.

**Table 6:** ANOVA results, showing: degrees of freedom (df), F-statistic values, and p-values for each factor.

Factor	Sum of Squares	df	F	p-value
Application	32064856.861017443	6.0	1.88009946080369	0.200467967609627
Kinematic Viscosity	545974.9761165365	1.0	0.19207706355744	0.67278695739922
Residual	22739830.19126059	8.0		

The multifactorial ANOVA analysis showed that differences in energy consumption between different oil applications are not statistically significant at the 0.05 level, and kinematic viscosity also does not have a significant impact on energy consumption. Therefore, neither variable has a significant effect on the dependent variable (energy consumption) in the analyzed model.

One of the key assumptions of the ANOVA test is the normality of the residuals. To verify this, the Shapiro-Wilk test was performed on the model’s residuals, yielding a Shapiro-Wilk statistic of 0.716 and a p-value of 0.00026. Since the p-value is significantly lower than the standard significance level of 0.05, we reject the null hypothesis of residual normality. This indicates that the residuals are not normally distributed, which may suggest that one of the assumptions of ANOVA is violated.

Due to this, a more robust analysis was subsequently conducted to account for the violation of the normality assumption. The Kruskal-Wallis test, a non-parametric alternative to ANOVA, was used since this test does not require normality of the data. It examines whether the differences between groups are statistically significant. The Kruskal-Wallis test results showed a test statistic of 7.56 and a p-value of 0.272. The result suggests that there are no statistically significant differences in energy consumption between groups with different oil applications (p-value > 0.05). Like the earlier ANOVA analysis, the Kruskal-Wallis test does not show significant differences between the groups.

## 4. Conclusions

The kinematic viscosity of oil influences the energy demand of a gear pump drive; however, this relationship is not dominant for modern oils used in vehicles and machinery. Differences in the operating conditions of oils used in internal combustion engines, transmissions, hydraulic systems, or two-stroke engine fuel mixtures lead to the addition of various agents that modify their structure. Consequently, this results in changes to the physical and chemical properties, making the energy consumption increase during the pumping of oils with higher kinematic viscosity less relevant in a broad analysis of oils for various applications, such as internal combustion engines, hydraulics, transmissions, shock absorbers, air conditioning systems, chainsaw lubrication, and two-stroke engines with premixed fuel systems. It can be assumed that this relationship holds within the scope of analyzing oils for a single application group. The difference in energy demand on the gear pump’s input shaft under recommended and effective operating conditions (gear pump speed of 2000 rpm and pressure of 20 MPa) amounted to 21%, ranging from 606 to 734 Wh. The lowest energy consumption was observed in the hydraulic oil that the manufacturer specifically recommended for use with the gear pump. Interestingly, this was not the oil with the lowest kinematic viscosity, likely due to the pump’s design being optimized for hydraulic oil HL 46. This research expands the understanding of gear pump energy consumption in relation to the pumping of oils used in machinery and vehicles across a wide range of kinematic viscosities at ambient temperatures, around 20°C, ranging from 21 to 784.5 mm^2^/s. Commercial oils available on the market were used in the study, although their precise compositions, especially regarding additives, are proprietary to the manufacturers, which limits the ability to draw precise conclusions about the causes of energy consumption variations. Gear pumps specifically designed for certain oil groups may yield different energy consumption results; however, one of the most popular types of gear pumps used in hydraulic systems was chosen for this study.
